# The emerging roles of protein homeostasis‐governing pathways in Alzheimer's disease

**DOI:** 10.1111/acel.12801

**Published:** 2018-07-10

**Authors:** Ji Cheng, Brian J. North, Tao Zhang, Xiangpeng Dai, Kaixiong Tao, Jianping Guo, Wenyi Wei

**Affiliations:** ^1^ Department of Gastrointestinal Surgery Union Hospital Tongji Medical College Huazhong University of Science and Technology Wuhan China; ^2^ Department of Pathology Beth Israel Deaconess Medical Center Harvard Medical School Boston Massachusetts

## Abstract

Pathways governing protein homeostasis are involved in maintaining the structural, quantitative, and functional stability of intracellular proteins and involve the ubiquitin–proteasome system, autophagy, endoplasmic reticulum, and mTOR pathway. Due to the broad physiological implications of protein homeostasis pathways, dysregulation of proteostasis is often involved in the development of multiple pathological conditions, including Alzheimer's disease (AD). Similar to other neurodegenerative diseases that feature pathogenic accumulation of misfolded proteins, Alzheimer's disease is characterized by two pathological hallmarks, amyloid‐β (Aβ) plaques and tau aggregates. Knockout or transgenic overexpression of various proteostatic components in mice results in AD‐like phenotypes. While both Aβ plaques and tau aggregates could in turn enhance the dysfunction of these proteostatic pathways, eventually leading to apoptotic or necrotic neuronal death and pathogenesis of Alzheimer's disease. Therefore, targeting the components of proteostasis pathways may be a promising therapeutic strategy against Alzheimer's disease.

## 1 INTRODUCTION

### 1.1 Alzheimer's disease

*Alzheimer's* disease (AD) is an age‐related neurodegenerative disorder clinically featuring loss of memory, cognitive, and behavior functions. In general, the severity of symptoms gradually worsens over time, which ultimately disables sufferers from daily tasks and activities (Wang, Gu, Masters, & Wang, 2017). As the most prevalent cause of dementia (accounting for nearly 70% of newly diagnosed dementia cases), *Alzheimer's* disease afflicted approximately 50 million patients globally in 2015 which is expected to rise to 115 million by the middle of 21st century, affecting half of the elderly population over 85 years old (Guo, Cheng, North, & Wei, 2017). Moreover, the number of early‐onset cases (those diagnosed with AD under the age of 65) has reached approximately 200,000 in the United States, indicating an intense urgency for better early identification methods and clinical management of *Alzheimer's* disease (Frisoni, Boccardi, Barkhof, Blennow, & Cappa, 2017; Guo, Cheng et al., 2017).

While AD has become the 6th leading cause of death for all Americans, there is still no effective therapeutic strategies against AD progression, primarily due to the lack of knowledge on its pathogenic mechanisms. At present, no medication is available to delay or halt disease progression. Instead, cholinesterase inhibitors (donepezil, galantamine, rivastigmine, and tacrine), NMDA (N‐methyl‐D‐aspartate) receptor antagonists (memantine), and some neural protective agents are utilized in a sole or synergistic manner to ameliorate the symptoms caused by neuronal loss, especially cognitive defects (Cummings, Aisen, DuBois, Frölich, & Jack, 2016). Therefore, a thorough understanding of the pathogenesis of AD is necessary in order for more effective and targeted therapeutics to be developed.

### 1.2 Pathological characteristics of Alzheimer's disease

In‐depth studies have proved that irreversible loss of neuronal cells in the cortical layer of hippocampus and medial temporal lobe is the most typical pathological alteration in patients with AD. Further discoveries have identified that there are two additional core subcellular hallmarks of *Alzheimer's* disease, termed the formation of senile plaques and neurofibrillary tangles, which are involved in the damage of neurons (Masters, Bateman, Blennow, Rowe, & Sperling, 2015). In particular, senile plaques are formed by the deposition of a protein fragment called amyloid beta (Aβ), which derives from the cleaved products of amyloid precursor protein (APP). The subsequent extracellular deposits of Aβ aggregates promote neuronal lethality as well as subsequent brain amyloidosis (Riek & Eisenberg, 2016). In addition, neurofibrillary tangles located inside the damaged neurons are formed by twisted fibers of tau protein. The hyperphosphorylation state of tau facilitates the formation of intracellular tangles, which directly disrupts neuronal homeostasis and culminates in aberrant cell death (Iqbal, Liu & Gong, 2016). These two major hallmarks are the most significant targets for AD research and therapeutics, either through inhibiting their formation or promoting disposal. Among those regulatory networks, a novel prolyl isomerase Pin1 has drawn much attention about its protective effects against neurodegeneration especially *Alzheimer's* disease (Liou, Sun, Ryo, Zhou, & Yu, 2003). With respect to physiology, Pin1 could directly inhibit the activity of GSK3β (glycogen synthase kinase‐3 β), which subsequently leads to reduced tau phosphorylation and the cleavage of APP into Aβ (Ma, Pastorino, Zhou, & Lu, 2012). Dysregulation of Pin1 has been identified in the pathogenesis of AD and thus recognized as a potential target for future therapeutics (Pastorino, Sun, Lu, Zhou, & Balastik, 2006).

In consideration of their central role in the pathogenesis of AD, pathological examination of Aβ plaques or tau tangles in the brain has been confirmed as the golden standard for the diagnosis of AD (Schaffer, Sarad, DeCrumpe, Goswami, & Herrmann, 2015). Furthermore, to simplify the clinical diagnosis, several peripheral biomarkers have already been chosen as supportive indicators, including the level of Aβ1‐42 and p‐tau181 within the CSF (cerebrospinal fluid) and the rate of glucose uptake by FDG‐PET (fluorodeoxyglucose‐positron emission tomography) (Ballard, Gauthier, Corbett, Brayne, & Aarsland, 2011; Cohen & Klunk, 2017; Khan & Alkon, 2015; Lista, Garaci, Ewers, Teipel, & Zetterberg, 2014). Moreover, other biomarkers are believed to have potential diagnostic values based on preliminary analyses, including ApoE4 level in the blood, YKL‐40 (chitinase‐3 like‐1) level in the CSF, and NTP (neuronal thread protein) level in the urine, despite insufficient confirmatory evidence (Craig‐Schapiro, Perrin, Roe, Xiong, & Carter, 2010; Farrer, 1997; Lonneborg, 2008). The combination of these diagnostic biomarkers could largely increase the accuracy and potential early diagnosis and thus benefit the prognosis of patients (Table 1).

**Table 1:** Major pathological biomarkers for diagnosis of *Alzheimer's* disease

Biomarker	Alterations	Detection methods	Diagnostic value	Prognosis
Aβ plaque	Elevated	Pathological examination	Golden standard (Schaffer et al., 2015)	Relevant (Pratico, 2013)
Tau tangles	Elevated	Pathological examination	Golden standard (Schaffer et al., 2015)	Relevant (Pratico, 2013)
Aβ1‐42	Decreased	CSF test	Supportive (Ballard et al., 2011; Lista et al., 2014)	Relevant (Tarawneh, D'Angelo, Crimmins, Herries & Griest, 2016)
p‐tau181	Elevated	CSF test	Supportive (Ballard et al., 2011; Khan & Alkon, 2015)	Relevant (Tarawneh et al., 2016)
Glucose uptake	Decreased	FDG‐PET	Supportive (Ballard et al., 2011; Cohen & Klunk, 2017)	Relevant (Silverman, Small, Chang, Lu & Kung De Aburto, 2001)
ApoE4	Decreased	Blood test	Potential (Farrer, 1997)	Relevant (Poirier, Delisle, Quirion, Aubert, & Farlow, 1995)
YKL‐40	Elevated	CSF test	Potential (Craig‐Schapiro et al., 2010)	Relevant (Craig‐Schapiro et al., 2010)
NTP	Elevated	Urine test	Potential (Lonneborg, 2008)	NS

*Note*

CSF: cerebrospinal fluid; FDG‐PET: fluorodeoxyglucose‐positron emission tomography; NS: not specific; NTP: neuronal thread protein; YKL‐40: chitinase‐3‐like‐1.

### 1.3 Prion‐like properties of Alzheimer's disease

The Prion disease is a rare neurodegenerative disorder with a pathological hallmark of PrP^Sc^ (PrP scrapie), an abnormal disease‐specific conformation of the prion proteins (Mead & Reilly, 2015). In contrast to other types of neurodegenerative diseases, the infectious and seeding ability is the most distinguishable feature of the Prion disease, which enables the spreading of misfolded prion proteins from peripheral regions to the central nervous system and even interspecies transmissibility (Victoria & Zurzolo, 2017).

In particular, recent investigations have confirmed that nonprion aggregates found in AD‐affected regions also share similar properties to prion protein that the pathogenic tau tangles and Aβ plaques could be transmitted by interneuronal connections and promote further disease pathogenesis (Yin, Tan, Jiang, & Yu, 2014). Specific to Aβ plaques, intercellular transfer is mediated by direct cellular connections. Further studies have discovered that the seeding of Aβ could not only occur between neurons but also happen between neurons and astrocytes, indicating a diversified mechanism of pathogenic spreading. Furthermore, accumulation of tau tangles is frequently detected among anatomically connected neurons. Subsequent in‐depth mechanistic study has clarified that the seeding of tau aggregates could be achieved by a trans‐synaptic mechanism in transgenic mouse models, which features an internalization of tau proteins localized at the membrane borders (Costanzo & Zurzolo, 2013). These results have implicated that blocking cell‐to‐cell structures and transmissions may be a potential therapeutic strategy for combating *Alzheimer's* disease.

### 1.4 Protein homeostasis‐governing pathways in regulation of Alzheimer's disease

#### 1.4.1 Protein homeostasis‐governing pathways

With respect to structure and function, proteins serve as the fundamental macromolecules for living cells, and therefore, their homeostasis is critical for the survival of organisms. The control of protein homeostasis occurs through either regulation of synthesis or degradation. For example, the mTOR pathway acts as a central pathway regulating protein synthesis, the activation of which accelerates the synthetic capability for cytoskeleton proteins and thus facilitates the cellular growth and proliferation (Saxton & Sabatini, 2017). On the other hand, a well‐balanced degradation of excessive or malformed proteins is also important to maintain protein homeostasis. Two major pathways regulating protein removal include autophagy and the ubiquitin–proteasome system (UPS) to promote the physiological degradation of marked proteins, either through a lysosome‐dependent or ubiquitination‐dependent manner (Leithe, 2016; Levy, Towers & Thorburn, 2017). Any aberrant event on these pathways can lead to protein dysregulation and result in pathological onset.

#### 1.4.2 Protein homeostasis‐governing pathways and *Alzheimer's* disease

**Figure 1:**
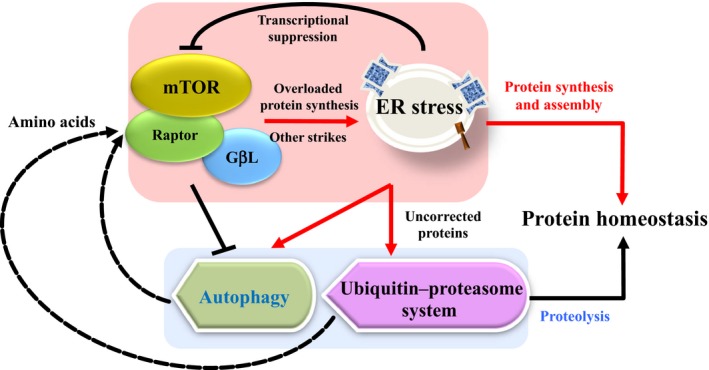
A schematic illustration of protein‐governing pathways in mammalian cells

Similar as other neurodegenerative disorders, the initiation and progression of AD are correlated to the accumulation of misfolded proteins, known as Aβ plaques and tau tangles that contribute to the progressive neuronal loss and cognitive impairment (Ciechanover & Kwon, 2015; DeToma, Salamekh, Ramamoorthy, & Lim, 2012). The fact that proteostasis pathways are essential for the production, modification, and clearance of intracellular proteins, thus its association with AD is intriguing even if expected. However, comprehensive review of the mechanistic landscape linking these protein homeostasis pathways with AD is lacking, and therefore, we have set out to summarize relevant information to provide mechanistic insights and guidance for future investigation (Figure 1).

In particular, there appear to be different pathological and biochemical features relevant to protein homeostasis between familial AD and sporadic AD. For example, it is reported that familial AD typically has an earlier age of onset, often under the age of 65, and mainly due to the mutations of *PSEN1*,* PSEN2,* and *APP* (1%–2% of total AD) (Wu, Rosa‐Neto, Hsiung, Sadovnick, & Masellis, 2012). Mutations within these genes (*PS1/PS2/APP*) affect a common pathogenic pathway in APP synthesis and proteolysis, which lead to excessive production of amyloid β, and the accumulation and deposition of amyloid β formed senile plaque acts as a major pathologic lesion of AD. Thus, new therapeutic agents targeting amyloid formation may benefit familial AD individuals. However, sporadic AD displays the pathological lesions including both the senile plaque and neurofibrillary tangle (NFT) with different mechanisms including the abnormal production or degradation of amyloid β and tau proteins as we summarized below. Thus, these protein homeostasis mechanisms may function differently to affect diverse pathological symptoms in familial vs. sporadic AD. In support of this notion, recent studies have demonstrated that familial AD bearing more mitochondrial unfolded protein response (mtUPR) gene activation (around 70%–90%) compared to sporadic AD (around 40%–60%), indicating that at least mtUPR is more relevant in pathogenesis of familial AD (Piras, Collin, Grüninger, Graff, & Rönnbäck, 2016).

## 2 PATHOLOGICAL CONTRIBUTIONS OF THE UBIQUITIN–PROTEASOME SYSTEM (UPS) IN *ALZHEIMER'S* DISEASE

### 2.1 General outline of the mammalian ubiquitin–proteasome system

The ubiquitin–proteasome system (UPS) is a highly conserved intracellular pathway to maintain protein homeostasis and critical during transcriptional activity, cell division, and stress responses (Cheng, Guo, Wang, North, & Tao, 2017; Kumari, Jaynes, Saei, Iyengar, & Richard, 2017). The proteins targeted for UPS‐mediated degradation are first ubiquitinated through an enzymatic cascade which tags the protein for recognition by the 26S proteasome for degradation (Kwon & Ciechanover, 2017).

The first step in the UPS cascade, an E1 enzyme, activates a 76‐amino‐acid polypeptide called ubiquitin through an ATP‐dependent manner, which successfully exposes the carboxyl group at the C‐terminus of ubiquitin, a binding site for target protein substrates. The activated ubiquitin is temporarily transferred to an E2‐conjugating enzyme via a transesterification reaction and a thioester bond formation at its cysteine residue. Following the E2 conjugation, the ubiquitin moiety then connects with an E3 ligase, which dictates the substrate specificity of protein substrates and functions to pass the ubiquitin moiety to a lysine residue on the target protein. In the end, 26S proteasome recognizes poly‐ubiquitinated proteins for eventual biochemical degradation (Ciechanover, 2015). Besides the canonical proteasome‐dependent degradation cascade, some ubiquitination events result in nondegradation consequences independent of proteasome pathway, such as subcellular relocalization or altered activity of protein substrates (Zheng, Zhou, Wang, & Wei, 2016).

### 2.2 The contribution of the ubiquitin–proteasome system to Aβ plaques formation

#### 2.2.1 The role of APP‐BP1 in the formation of Aβ plaques

Neddylation is a highly conserved process analogous to ubiquitination, in which specific E1, E2, and E3 enzymes constitute a post‐translational modification cascade to facilitate the conjugation of ubiquitin‐like protein NEDD8 (neural precursor cell expressed, developmentally downregulated 8) to targeted proteins (Enchev, Schulman & Peter, 2015). All of the cullin scaffolds within Cullin‐RING ligases (CRLs) share a similar cullin homology domain and carboxyl‐terminal neddylation site, thus becoming the largest substrate family of neddylated substrates, although there are still many noncullin substrates of neddylation E3 enzymes such as p53, Histone H4, and the ribosomal protein L11. A consequence of cullin scaffold neddylation is an enhanced ubiquitinating activity of the CRL as a result of conformational changes that occur during the neddylation process (Brown & Jackson, 2015).

Amyloid‐β precursor protein‐binding protein 1 (APP‐BP1) was first identified in 1996 as a novel protein that bound to the carboxyl‐terminal region of amyloid‐β precursor protein (APP) (Chow, Korenberg, Chen, & Neve, 1996). Structural analysis has confirmed that APP‐BP1 is one of the two subunits that constitute a heterodimeric NEDD8‐activating enzyme (NAE). Comparable to ubiquitin activation, NAE is crucial for the initiation of a neddylation cascade due to its capability to expose and activate the C‐terminus of NEDD8 (Enchev et al., 2015). Therefore, APP‐BP1 acts as a core stimulator of the ubiquitin–proteasome system as it can promote the activation of CRLs by playing the role of a neddylation E1 enzyme and initiating NEDD8 transfer.

**Figure 2:**
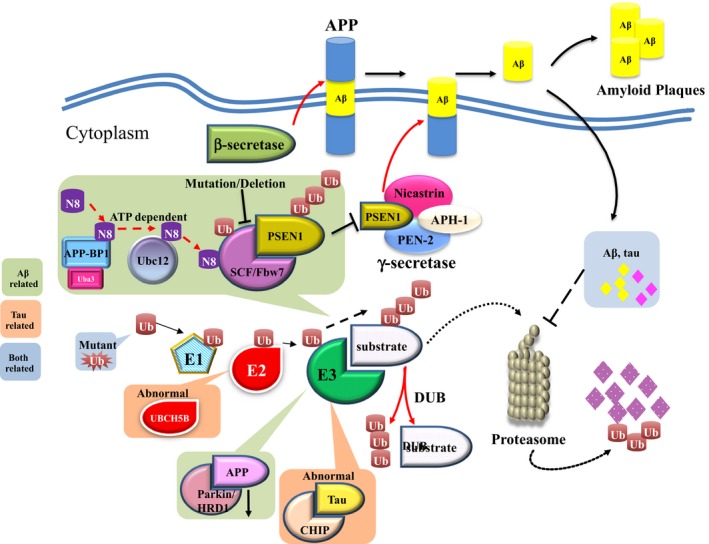
Mechanistic correlations between ubiquitin–proteasome system and *Alzheimer's* disease. N8: NEDD8; UB: ubiquitin

Involvement of APP‐BP1 and the formation of Aβ plaques have been proposed (Chen, Liu, McPhie, Hassinger, & Neve, 2003). It is well known that APP undergoes γ‐secretase cleavage to produce Aβ. However, to some extent, the quantity and activity of γ‐secretase determine the production of Aβ peptide and the severity of AD (Guo, Cheng et al., 2017). Mechanistic evidence has revealed that APP‐BP1‐involved neddylation of Cullin 1 and activation of CRL1 complex culminate in Presenilin‐1 degradation, which is an enzymatic component of the γ‐secretase complex (Chen, Neve & Liu, 2012). These regulatory mechanisms suggest that when the expression level of APP‐BP1 is elevated, the levels of Aβ would decrease accordingly. Nevertheless, this idea remains controversial with the observation that APP‐BP1 expression has been shown to increase in AD‐affected regions of the brain (Chen et al., 2003). In an important way, overexpression of APP‐BP1 has been shown to trigger cytoplasmic relocalization of NEDD8 within neuronal cells, which may erroneously incorporate into the ubiquitin chains of degradation substrates and disrupt the subsequent proteolytic activities instead of enhancing the ubiquitination activity (Chen et al., 2012). These reports have revealed a potential contributory role of dysfunctional neddylation to the pathogenesis of Aβ plaque formation, and however, further investigation remains necessary to clarify the underlying mechanisms (Figure 2).

#### 2.2.2 Other pathogenic correlations between ubiquitin–proteasome system and Aβ plaque formation

Under normal circumstances, the ubiquitin–proteasome system serves as a major regulator of Aβ accumulation in neuronal cells, either by decreasing the production of Aβ or promoting its proteolytic degradation (Hong, Huang & Jiang, 2014). Therefore, any dysregulation in the ubiquitin–proteasome system may lead to Aβ accumulation in the cytoplasm of neurons which facilitates the formation of Aβ plaque and consequently cell death.

Additional studies have revealed that decreased expression of the ubiquitin E3 ligases Parkin and HRD1 (E3 ubiquitin protein ligase HRD1) is a common observation in AD specimens, which reduces the degradation of APP thereby increased the amount of its cleaved Aβ (Nomura, Hosoi, Kaneko, Ozawa, & Nishi, 2016). On the contrary, accumulated Aβ may down regulate proteasomal activity and subsequently result in malfunction of the multivesicular bodies sorting pathway, which further blocks the degradation of Aβ itself, forming a vicious cycle that contributes to the formation of pathogenic plaques (Hong et al., 2014; Tramutola, Domenico, Barone, Perluigi, & Butterfield, 2016). Apart from this major mechanistic interpretation, there are still other mechanisms that might explain the association between the UPS and Aβ, such as mutant ubiquitin, which has been detected genetically in certain AD patients with impaired ubiquitination activity and thus results in the accumulation of Aβ plaques (Lam, Pickart, Alban, Landon, & Jamieson, 2000).

Taken together, although several studies have speculated that oxidative damage on UPS components may be a vital initiation factor for Aβ accumulation (Tramutola et al., 2016), the majority of evidences still hint that ubiquitin–proteasome system may play a crucial role in the formation of Aβ plaques.

### 2.3 Tau aggregation and the ubiquitin–proteasome system

Tau is an axonal cytosolic protein serving to stabilize the microtubule network inside neurons. Various post‐translational modifications appear to affect the activity of tau in diverse contexts, among which the phosphorylation on tau protein is critically highlighted (Iqbal et al., 2016). Tau phosphorylation is reduced during embryogenesis, resulting in increased neuronal plasticity in the neonatal nervous systems (Wang & Mandelkow, 2016). Multiple kinases are capable of inducing tau phosphorylation, including GSK3β, CDK5 (cyclin‐dependent kinase 5), PKA (cAMP‐dependent protein kinase), and MAPK1 (mitogen‐activated protein kinase 1). Aberrant hyperphosphorylation of tau weakens its interaction with microtubules leading to destabilization of the structure of microtubule as well, which therefore facilitates the formation of tau tangles and damages the neuronal homeostasis (Lee, Lee & Rubinsztein, 2013; Tarasoff‐Conway, Carare, Osorio, Glodzik, & Butler, 2015).

The ubiquitin–proteasome system functions as a key pathway for tau clearance, with a specific enzymatic cascade contributing to the ubiquitination and degradation of tau. Similar to Aβ formation, any dysregulation of the ubiquitin–proteasome system could halt the degradation of tau protein and, in combination with hyperphosphorylation, promote the formation of tau tangles (Lee et al., 2013). At present, there are two characterized mechanisms that lead to defective degradation on tau. First, as described above, cytoplasmic NEDD8 induced by APP‐BP1 overexpression triggers erroneous incorporation of NEDD8 into polyubiquitin chains, which interferes with the normal degradation process and results in excessive storage of tau in neurons (Chen et al., 2012). Second, impairment on proteasomal activity within susceptible neurons may also promote the aggregation of tau, whose dysfunction may be either attributed to age‐related insufficiency or the proteotoxic effect of tau aggregates (Lee et al., 2013; Myeku, Clelland, Emrani, Kukushkin, & Yu, 2016). Other mechanisms may involve the dysfunction of the tau‐specific E2 enzyme UBCH5B (ubiquitin‐conjugating enzyme E2 D2, alias UBE2D2), and E3 ligase CHIP (carboxyl‐terminus of Hsc70 interacting protein) might also accelerate the accumulation of tau protein, although mechanistic details largely remain unclear (Saidi, Polydoro, Kay, Sanchez, & Mandelkow, 2015; Shimura, Schwartz, Gygi, & Kosik, 2004). Moreover, considering hyperphosphorylation as a primary mechanism of tau tangle formation, the potential association between ubiquitin–proteasome system and tau‐related kinases will be an important area of investigation in the future, due to the mutual crosstalk among ubiquitin–proteasome system, tau protein, and tau‐related kinases (Table 3).

### 2.4 Alzheimer's disease‐relevant phenotypes in genetic mouse models of components of the ubiquitin–proteasome system

Considering potential contributions of the UPS in regulating AD pathogenesis, specific knockout mouse models have been generated to elucidate the phenotypic features secondary to genetic alterations on certain UPS components. Parkin acts as a vital E3 ligase involved in the degradation of APP within neurons. Systemic deletion of *Parkin* results in viable offspring with normal brain morphology. However, these animals gradually develop behavioral defects and neuronal cell death (Goldberg, Fleming, Palacino, Cepeda, & Lam, 2003). In addition, the ubiquitin ligase UBE3A (ubiquitin protein ligase E3A) destabilizes p53, the heterozygous ablation of which causes significant memory loss and dementia phenotypes among genetically engineered mice (Jiang, Armstrong, Albrecht, Atkins, & Noebels, 1998). CHIP, a tau protein E3 ligase, functions to clear phosphorylated tau in neurons, and deletion of *CHIP* correspondingly results in the accumulation of hyperphosphorylated tau protein and facilitates the progression of AD (Dickey, Yue, Lin, Dickson, & Dunmore, 2006). In addition, FBW7, a constitutive substrate receptor for Cullin 1‐RING E3 ligases, displays critical involvement in neural development as its conditional knockout in neural stem cells leads to impaired differentiation and neuronal loss (Wang, Inuzuka, Fukushima, Wan, & Gao, 2011).

Apart from E3 ligases, DUBs have also been highlighted to be important for AD pathogenesis as revealed by knockout mouse models of DUBs such as UCHL1 (ubiquitin C‐terminal hydrolase L1) and UCHL3 (ubiquitin C‐terminal hydrolase L3). Both DUBs are abundantly expressed in the central nervous system while the genetic loss of either protein leads to severe memory loss, axonal dystrophy, and other neurodegenerative phenotypes (Kurihara, 2001; Wood, Kaplan, Brensinger, Guo, & Abel, 2005). This may be mechanistically attributed to the fact that loss of UCHL1 and UCHL3 accelerates the degradation of APP, thus accounting for the overproduction of Aβ (Zhang, Cai, Zhang, Zhang, & Song, 2014) (Table 2).

**Table 2:** Major *Alzheimer's* disease‐relevant phenotypes of genetic mouse models on components of protein‐governing machineries

Machinery	Gene	Alteration	Phenotype
Ubiquitin–proteasome system	*Parkin* ^*−/−*^	Systemic knockout	Behavioral defects and neuronal death (Goldberg et al., 2003)
	*Uchl1* ^*−/−*^	Systemic knockout	Axonal dystrophy and neurodegeneration (Yamazaki, Wakasugi, Tomita, Kikuchi, & Mukoyama, 1988)
	*Uchl3* ^*−/−*^	Systemic knockout	Neurodegeneration (dystrophic axons and impaired memory) (Kurihara, 2001; Wood et al., 2005)
	*Ube3a* ^*+/−*^	Systemic knockout	Learning and memory deficits (Jiang et al., 1998)
	*Mgrn1* ^*−/−*^	Systemic knockout	Neurodegeneration (He, Lu, Jolly, Eldridge, & Watson, 2003)
	*Chip* ^*−/−*^	Systemic knockout	Accumulation of hyperphosphorylated tau protein (Dickey et al., 2006)
	*Fbw7* ^*−/−*^	Conditional knockout (neural stem cell)	Impaired differentiation into neurons and neonatal fatality (Wang et al., 2011)
Autophagy	*Ambra1* ^*+/−*^	Systemic knockout	Impaired neurogenesis and enhanced neuronal apoptosis (Yazdankhah et al., 2014)
	*Atg5* ^*−/−*^	Conditional knockout (neural cells)	Neurodegeneration (motor function disorder and accumulation of inclusion bodies) (Hara et al., 2006)
		Conditional knockout (Purkinje cells)	Accumulation of aberrant membrane structures and neuronal death (Nishiyama et al., 2007)
	*Atg5*	Transgenic	Anti‐aging phenotypes (better insulin sensitivity and motor function) (Pyo et al., 2013)
	*Atg7* ^*−/−*^	Conditional knockout (neural cells)	Neurodegeneration (massive neuronal loss, behavioral defects, neonatal lethality) (Komatsu et al., 2006; Komatsu, Waguri et al., 2007); intracellular Aβ accumulation (Nilsson et al., 2013)
		Conditional knockout (Purkinje cells)	Progressive dystrophy and degeneration of the axon terminals (Komatsu, Wang, Holstein, Friedrich, & Iwata, 2007)
	*Atg17* ^*−/−*^	Conditional knockout (neural cells)	Cerebellar degeneration (progressive neuronal loss, spongiosis, neurite degeneration, and accumulation of ubiquitinated protein aggregates) (Liang et al., 2010)
	*Beclin1* ^*+/−*^	Systemic knockout	Increased neuronal apoptosis (Yazdankhah et al., 2014); accumulation of Aβ (Pickford et al., 2008)
	*Ulk1* ^*−/−*^	Conditional knockout (neural cells)	Neurodegeneration (neuronal loss) (Joo et al., 2016)
	*Ulk2* ^*−/−*^	Conditional knockout (neural cells)	Neurodegeneration (neuronal loss) (Joo et al., 2016)
mTOR signaling	*mTOR*	Transgenic (forebrain)	Cortical atrophy and neurodegeneration (Kassai et al., 2014)
	*Tsc1* ^*−/−*^	Conditional knockout (forebrain)	Brain abnormalities and neurodegeneration (Carson et al., 2012)
ER stress	*Ire1* ^*−/−*^	Conditional knockout (brain)	Less cognitive impairment (Duran‐Aniotz et al., 2017)

*Note*

Aβ: amyloid beta; ER: endoplasmic reticulum.

### 2.5 Therapeutic implications

The poor prognosis of AD and the ineffectiveness of therapeutic strategies, with more than 200 therapeutic interventions currently under clinical trials, highlights the therapeutic dilemma at present and the urgency to identify novel therapeutic targets (Gadhave, Bolshette, Ahire, Pardeshi, & Thakur, 2016). With the mechanisms described above in minds, the main principle for the design of UPS‐targeted therapeutics would be to boost its proteolytic impact on pathogenic substances, including Aβ plaques and tau tangles. As the proteasome is the core element within the ubiquitin–proteasome machinery and the impairment of proteasome activity is commonly observed in AD cases, most of the therapeutics that specifically targets the ubiquitin–proteasome system has been concentrated on regulating proteasome activation (Ciechanover & Kwon, 2015; Gadhave et al., 2016).

Thus far, only resveratrol has progressed into Phase III clinical trials as a therapeutic candidate for AD, which restores proteasomal activity and exhibits anti‐amyloidogenic effects on susceptible neurons (Marambaud, Zhao & Davies, 2005; Turner, Thomas, Craft, Dyck, & Mintzer, 2015). Other preclinical drugs with potential efficacy toward AD include betulinic acid, IU1, and proteasome‐activating peptide 1. Betulinic acid is a lupine‐type pentacyclic triterpene that enhances the chymotrypsin‐like activity of proteasomes (Navabi, Sarkaki, Mansouri, Badavi, & Ghadiri, 2018). IU1 is an enzyme that counteracts the function of proteasome‐associated USP14, promoting the degradation of the tau protein (Kiprowska, Stepanova, Todaro, Galkin, & Haas, 2017). In in vivo studies, proteasome‐activating peptide 1 elevates the chymotrypsin‐like activity of the proteasome and antagonizes the destructive influence of aberrant plaques and tangles (Gadhave et al., 2016). These potential therapeutics indicate that UPS‐targeted agents are a promising area of development for anti‐AD strategies in the near future (Table 4).

## 3 PATHOLOGICAL ASSOCIATION BETWEEN AUTOPHAGIC PATHWAYS AND *ALZHEIMER'S* DISEASE

### 3.1 Autophagic machinery

Autophagy is an evolutionally conserved protein homeostasis‐governing pathway in mammalian cells (Min, Choy, Pollock, Tu, & Hornicek, 2017). In brief, autophagosomes with engulfed substances migrate toward intracellular lysosomes along the microtubule cytoskeleton, where they then fuse with the lysosomes and undergo lysosomal proteolysis. According to the contents of engulfed substances to be degraded, such as misfolded proteins, redundant peroxisomes, pathogenic organisms, and dysfunctional mitochondria, the autophagic machinery has been subdivided into four subtypes, namely aggrephagy, perophagy, xenophagy, and mitophagy, respectively (Cordani, Butera, Pacchiana, & Donadelli, 2017; Guo, Cheng et al., 2017; Kaur & Debnath, 2015). The autophagic pathway utilizes a well‐orchestrated cascade of forming and targeting autophagosomes carried out by autophagy‐related proteins (ATGs) (Levy et al., 2017). Under physiological conditions, the autophagic cascade is initiated following the activation of AMPK by energy deprivation, which consequently exerts inhibitory effects on mTORC1 (mTOR complex 1) (Cadwell, 2016; Cheng, Zhang, Ji, Tao, & Guo, 2016). Next, the activity of the transcription factor EB (TFEB) is enhanced as a result of mTORC1 inhibition, facilitating the transcriptional induction of autophagy‐relevant genes, including Atg5, Atg12, and Atg16 (Noda, 2017). Meanwhile, the repression of mTORC1 activity stimulates the kinase activity of ULK1 (unc‐51 like autophagy activating kinase 1), which then activates its downstream target Vps34. Vps34 synthesizes a product called PI3P (phosphatidylinositol 3‐phosphate), which is inevitable for the recruitment of Atg12‐Atg5‐Atg16 hetero‐trimer to the phagopore (Guo, Cheng et al., 2017). The membrane elongation and cargo recognition process require the involvement of this trimeric complex, as well as an ubiquitin‐like protein LC3 (microtubule‐associated protein 1 light chain 3). An autolysosome is then formed by the fusion of autophagosome and the lysosome, leading to the degradation of the cargo within the autophagosome (Guo, Cheng et al., 2017; Levy et al., 2017).

### 3.2 Aberrant autophagy in the formation of Aβ plaques and tau aggregates

Deficiency in autophagy is a commonly observed pathophysiological alteration in AD‐affected neurons, typically showing accumulation of immature autophagic vacuoles at neuronal dendrites (Li, Liu & Sun, 2017). Possible mechanisms may be that the expression of autophagy‐related genes decreases with (Omata, Lim, Akao, & Tsuda, 2014), as well as ApoE4 induces membrane disruption of lysosomes, release of lysosomal enzymes, leading to dysfunction of autophagic machinery (Ji, Müllendorff, Cheng, Miranda, & Huang, 2006).

Compared to the ubiquitin–proteasome system, autophagy is another major scavenger against Aβ redundancy, via degrading the Aβ peptide directly, degrading APP to reduce the production of Aβ, or triggering the extracellular secretion of Aβ (Li et al., 2017). Therefore, in the setting of autophagic dysfunction during AD progression, Aβ is stockpiled in neuronal cytoplasm. Moreover, another result of excessive Aβ production is that Aβ is detected within the immature autophagic vacuoles. This may be possibly owing to the pathological co‐localization of APP and β‐secretase in those vacuoles, as well as adequate reaction time due to the halted maturation of vacuoles, as both APP and β‐secretase are transported by vacuoles under physiological conditions (Das, Scott, Ganguly, Koo, & Tang, 2013; Li et al., 2017).

Similar as that of Aβ, autophagy is also regarded as an alternative pathway for tau clearance in addition to the ubiquitin–proteasome system (Li et al., 2017). Defective autophagy may not only disrupt degradation induced by the NRF2‐NDP52 (calcium binding and coiled‐coil domain 2) axis (Jo, Gundemir, Pritchard, Jin, & Rahman, 2014), but also enhance the hyperphosphorylation of tau through an unspecified mechanism (Inoue, Rispoli, Kaphzan, Klann, & Chen, 2012). Furthermore, hyperphosphorylated tau protein is found to further impair autophagy (Lim, Hernández, Lucas, Gómez‐Ramos, & Morán, 2001). This is mainly because hyperphosphorylation of tau may destabilize and disassemble the microtubule cytoskeleton, which subsequently blocks the retrograde trafficking of autophagosomes and causes accumulation of immature autophagosomes in axons (Li et al., 2017).

**Figure 3:**
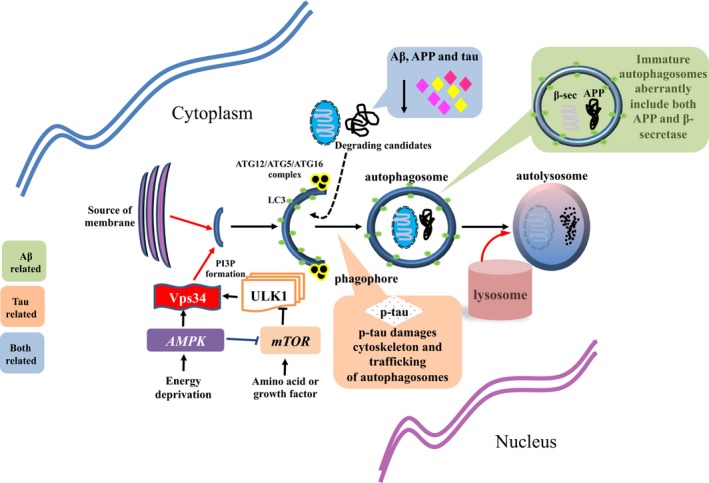
Pathological associations between mammalian autophagy machinery and *Alzheimer's* disease

Taken together, the mutual interactions between aberrant autophagy and AD pathogenesis seem to form a vicious cycle that critically contributes to the onset of AD. However, which one is the initiating factor remains unclear, and therefore, further studies are necessary to clarify this important question (Table 3) (Figure 3).

**Table 3:** Key functions of protein homeostasis‐governing pathways in *Alzheimer's* disease

Machinery	Role in *Alzheimer's* disease		
	Component	Alteration	Functional consequences
Ubiquitin–proteasome system	Neddylation	Upregulation	Elevated formation of Aβ plaques; excessive storage of tau protein
	E3 ligase	Downregulation	Reduced degradation of APP
	26S proteasome	Impaired	Defective proteolytic activity against tau accumulation

	Alteration	Tau aggregates	Aβ plaques
Autophagy	Autophagic insufficiency	Decreased removal and increased hyperphosphorylation on tau protein	Reduced degradation of Aβ, APP, and enhanced production of Aβ
mTOR signaling	Aberrantly activated	Inhibition of autophagic clearance on tau aggregates and activation of kinases of tau proteins to induce hyperphosphorylation	Reduced removal of excessive Aβ due to oxidative stress induced mTOR activation and subsequent autophagic inhibition
ER	Enhanced	UPR signaling directly activates tau phosphorylation	Increased APP‐Aβ transition

*Note*

APP: amyloid precursor protein; UPR: unfolded protein response.

### 3.3 Major Alzheimer's disease‐relevant phenotypes of genetic mouse models on components of autophagy pathway

Given the critical role that autophagy plays in the development of AD, multiple knockout mouse models have been established to elucidate the functions of specific targets inside the autophagic machinery. In brief, haplo‐insufficiency of Beclin1 and Ambra1, both proteins contributing to the assembly of autophagosomes, leads to neuronal apoptosis, accumulation of Aβ, and impaired neurogenesis (Pickford, Masliah, Britschgi, Lucin, & Narasimhan, 2008; Yazdankhah, Farioli‐Vecchioli, Tonchev, Stoykova, & Cecconi, 2014). Depletion of *ATG5*, a constitutive component of Atg12‐Atg5‐Atg16 hetero‐trimer that facilitates the elongation of phagopores, also corresponds to neurodegenerative phenotypes including neuronal death and accumulation of inclusion bodies (Hara, Nakamura, Matsui, Yamamoto, & Nakahara, 2006; Nishiyama, Miura, Mizushima, Watanabe, & Yuzaki, 2007). On the other hand, transgenic overexpression of *Atg5* displays anti‐aging consequences, ameliorating insulin sensitivity, and motor function in the central nervous system (Pyo, Yoo, Ahn, Nah, & Hong, 2013). Meanwhile, other essential autophagy‐related proteins such as Atg7 and Atg17 likewise demonstrate significant impacts toward neural homeostasis and AD progression. In particular, conditional deletion of *Atg7* results in massive neuronal death, behavioral impairments, and accumulation of intracellular Aβ (Komatsu et al., 2006; Komatsu, Wang et al., 2007; Nilsson, Loganathan, Sekiguchi, Matsuba, & Hui, 2013), while *Atg17*‐null neuronal cells suffer progressive loss and aggregation of proteotoxins as well (Liang, Wang, Peng, Gan, & Guan, 2010). Moreover, conditional deficiency of *Ulk1* in the central nervous system also results in neuronal loss and degeneration, which normally serves as a key kinase triggering the formation of PI3P (Joo, Wang, Frankel, Ge, & Xu, 2016) (Table 2). Overall, all these evidence confirm that dysregulation of autophagy may closely and positively correlate to the pathogenesis of AD.

### 3.4 Pharmaceutical progresses

It is well demonstrated that the mTOR pathway is able to repress the activity of autophagic machinery under physiological circumstances. Thus, the majority of autophagy‐targeted therapies aim to inhibit mTOR activity and then revive the autophagic clearance of misfolded proteins in AD susceptible neurons. Rapamycin is a classical mTOR inhibitor which has shown anti‐AD efficacy, which decreases fibrillary tangles and Aβ plaques in brain cells and restores cognitive impairment (Caccamo, Majumder, Richardson, Strong, & Oddo, 2010). Analogs of rapamycin, such as temsirolimus, have also demonstrated anti‐AD efficacy (Jiang, Yu, Zhu, Tan, & Wang, 2014). Furthermore, there is another mTOR‐dependent autophagy activator, arctigenin, which is a natural extract from Arctium lappa and accelerates the clearance of Aβ plaques (Zhu, Yan, Jiang, Yao, & Chen, 2013).

In terms of mTOR‐independent mechanisms for autophagy restoration, activation of AMPK (AMP‐activated protein kinase) usually becomes the optimal pharmaceutical solution, such as the use of trehalose and metformin (Menzies, Fleming, Caricasole, Bento, & Andrews, 2017). Trehalose, a disaccharide that inhibits glucose transporters, leads to the activation of AMPK and autophagy, attenuating the proteotoxic effects in affected neurons (DeBosch, Heitmeier, Mayer, Higgins, & Crowley, 2016; Menzies et al., 2017). Metformin, another well‐known AMPK activator initially approved to treat patients with diabetes, could also restore the machinery of autophagy and exhibit anti‐AD effects (Levy et al., 2017). In addition, GTM‐1, a novel small molecule, can ameliorate neurotoxicity triggered by oligomeric Aβ accumulation through an mTOR‐independent activation of autophagy (Guo, Liu, Cai, & Le, 2017) (Table 4).

**Table 4:** Major targeted drugs of protein‐governing pathways against *Alzheimer's* disease

Machinery	Drug	Target	Pharmacological mechanism	Stage
Ubiquitin–proteasome system	Resveratrol	Proteasome	Restoring proteasomal activity against Aβ accumulation (Ma, Tan, Yu, & Tan, 2014)	Ongoing clinical trial
	Betulinic acid	Proteasome	Enhancing the chymotrypsin‐like activity of proteasome (Navabi et al., 2018)	Preclinical assessment completed
	IU1	Proteasome	Catalyzing the enzymatic stimulation of proteasomal activity against redundant tau protein (Kiprowska et al., 2017)	Preclinical assessment completed
	PAP1	Proteasome	Stimulating proteasome as well as protecting against the destructive influences by plaques and tangles (Gadhave et al., 2016)	Preclinical assessment completed
Autophagy (mTOR)	Rapamycin	mTOR	A mTOR inhibitor that decreases formation of tangles and plaques by activating autophagic activity (Zhang, Wang, Wang, Gao, & Che, 2017)	Preclinical assessment completed
	Temsirolimus	mTOR	A rapalog that mimics the functional mechanism of rapamycin (Jiang et al., 2014)	Preclinical assessment completed
	Arctigenin	mTOR	A natural extract from *Arctium lappa* that activates autophagic pathway in a mTOR‐dependent manner (Zhu et al., 2013)	Preclinical assessment completed
	Trehalose	AMPK	A disaccharide that inhibits glucose transporters leads to the activation of AMPK and autophagic system (Du, Liang, Xu, Sun, & Wang, 2013)	Preclinical assessment completed
	Metformin	AMPK	A well‐known AMPK activator that enhances the activity of autophagic machinery (DiTacchio, Heinemann & Dziewczapolski, 2015)	Clinical trial completed
	GTM‐1	Autophagy	A mTOR‐independent small molecule for autophagic activation (Guo, Liu et al., 2017)	Preclinical assessment completed
ER stress	4‐phenylbutyric acid	ER folding process	A molecular chaperone that improve the folding ability of ER and thus restricts ER stress (Ricobaraza, Cuadrado‐Tejedor, Marco, Pérez‐Otaño, & García‐Osta, 2012)	Ongoing clinical trial
	Tauroursodeoxycholic acid	ER folding process	A molecular chaperone that improve the folding ability of ER and thus restricts ER stress (Nunes, Amaral, Lo, Fonseca, & Viana, 2012)	Ongoing clinical trial
	Trimethylamine oxide	ER folding process	A molecular chaperone that improve the folding ability of ER and thus restricts ER stress (Kraskiewicz & FitzGerald, 2012)	Preclinical assessment completed
	Dantrolene	PERK	Inhibition of ER calcium release and subsequent activation of PERK (Peng, Liang, Inan, Wu, & Joseph, 2012)	Preclinical assessment completed
	Edavorene	PERK	An inhibitor of PERK to ameliorate proteotoxic reactions (Placido, Pereira, Duarte, Candeias & Correia, 2014)	Preclinical assessment completed
	GSK2606414	PERK	An inhibitor of PERK to ameliorate proteotoxic reactions (Radford et al., 2015)	Preclinical assessment completed
Others	PU‐DZ8	HSP90	A HSP90 inhibitor that reduces the amount of aggregated and hyperphosphorylated tau (Zhao, Michaelis & Blagg, 2012)	Preclinical assessment completed
	YM‐08	HSP70	A HSP70 activator that limits the accumulation of pathogenic tau in brains (Lu, Tan, Wang, Xie, & Yu, 2014)	Preclinical assessment completed

*Note*

PAP1: proteasome‐activating peptide 1.

## 4 NECROSIS: AUTOPHAGY‐RELATED CELL DEATH THAT CONTRIBUTES TO THE NEURONAL DEATH OF *ALZHEIMER'S* DISEASE

There are two types of cell death for mammalian cells, namely apoptosis and necrosis (Chen, Yu & Zhang, 2016). Apoptosis is a programmed cell death with controllable intracellular mechanisms, pathologically featuring maintenance of ATP levels and condensed nuclei inside shrunken cells as the consequences of activated caspase cascades (Ichim & Tait, 2016; Nagata & Tanaka, 2017), whereas necrosis has been regarded as an acute type of cell death, which is characterized by ATP deprivation, swollen cytoplasm, and cell lysis. In contrast to apoptosis, necrosis is a rapid cellular reaction without programmed procedures, which makes it more difficult to control or reverse by therapeutic intervention (Gorman, 2008; Nikolova, Roos, Krämer, Strik, & Kaina, 2017).

In general, elevated intracellular concentration of calcium ions is an essential trigger of necrotic cell death as the result of severe stresses, either via membrane channel transport or release from subcellular organelles. Calcium ions can activate calpain proteases in the cytoplasm, which subsequently destroy the integrity of lysosomes and unleash hydrolytic enzymes such as cathepsin proteases. These events synergistically induce cell swelling, cell lysis, and eventually cell death (Green & Llambi, 2015; Troulinaki & Tavernarakis, 2012). Recent studies have identified an additional type of programmed cell death that could be regulated and controlled at the same time sharing the pathological hallmarks of necrosis, which is regulated necrosis or necroptosis (Weinlich, Oberst, Beere, & Green, 2017). As a subtype of necrosis, necroptosis was initially observed following cellular inflammatory events. The intracellular machinery that activates necroptosis is composed of three key components, namely RIPK1 (receptor‐interacting serine/threonine kinase 1), RIPK3 (receptor‐interacting serine/threonine kinase 3), and MLKL (mixed lineage kinase domain‐like pseudokinase) (Caccamo, Branca, Piras, Ferreira, & Huentelman, 2017; Chen et al., 2016). RIPK1 and RIPK3 are essential components that form necrosomes ahead of the activation of necroptosis. The formation of necrosomes triggers necroptosis by activating and phosphorylating MLKL, which culminates in mitochondrial uncoupling, lipid peroxidation, and cell death (Caccamo et al., 2017).

### 4.1 Necrotic neuronal death in AD progression and its clinical significances

Besides the inevitable role that apoptosis plays in the induction of neuronal death, necrosis also significantly contributes to cell survival in AD‐affected regions, especially under extremely severe situations, acting as the final and deadly response against proteotoxic impacts (Yamashima, 2016). In an mechanistic manner, the accumulation of Aβ and tau proteins triggers cytoplasmic increase in calcium ions to activate calpain proteases, which then cleaves the carbonylated HSP70.1 and leads to instability of lysosomal membranes. The rupture of lysosomes releases cathepsin proteases to eventually induce neuronal death. Meanwhile, failure of autophagy in neurons is also able to initiate the calpain–cathepsin‐dependent necrotic pathway, partially accounting for the loss of neurons during AD progression (Yamashima, 2013, 2016). On the other hand, necroptosis likewise contributes to neuronal death of *Alzheimer's* disease. Enhanced activity of RIPK1 is observed in AD‐affected regions, and lowering the activation of necroptosis has been shown to reduce cell loss in mouse models (Caccamo et al., 2017). However, mechanisms that induce the activation of necroptosis remain unclear.

As necrosis is mechanistically uncontrollable, necroptotic components therefore gain much attention on their potential therapeutic potential. At present, the inhibitors of RIPK1, such as Necrostatin‐1, display efficacy against multiple diseases, including *Alzheimer's* disease, immune‐dependent hepatitis, and glaucoma (Do, Sul, Jang, Kang, & Kim, 2017; Le Cann, Delehouzé, Leverrier‐Penna, Filliol, & Comte, 2017; Ofengeim, Mazzitelli, Ito, DeWitt, & Mifflin, 2017). In particular, the administration of RIPK1 inhibitors significantly decreases amyloid burden and cognitive defects among transgenic mice, suggesting its potential for future clinical development (Ofengeim et al., 2017).

### 4.2 Correlation between necrosis and autophagy in AD

At present, a correlation between autophagy and necrosis has attracted much attention due to their key participation in AD pathogenesis. As mentioned above, autophagy functions as a protective cellular event for neurons against numerous insults, such as oxygen scavengers and excessive proteotoxins, which eventually prevents the onset of necrotic signaling (Eisenberg‐Lerner & Kimchi, 2012). Nonetheless, in the setting of *Alzheimer's* disease, dysregulation of autophagy has diminished its protective impacts against lethal insults, thus favoring neuronal necrosis (Nikoletopoulou, Markaki, Palikaras, & Tavernarakis, 2013).

Despite a largely unclear mechanism, PARP (poly(ADP‐ribose) polymerase 1)‐mediated signaling is believed to facilitate the interconnection between autophagy and necrosis (Chaabane, User, El‐Gazzah, Jaksik, & Sajjadi, 2013; Los, Mozoluk, Ferrari, Stepczynska, & Stroh, 2002). The activation of PARP either promotes transcription of autophagy components or induces ATP depletion and subsequent necrotic cell death. The balance between the two paths is crucial to dictate the final outcome, and therefore, when autophagy is dysregulated in neurons, PARP activation favors irreversible necrotic cell death (Nikoletopoulou et al., 2013).

## 5 THE IMPORTANT ROLE OF PROTEIN SYNTHESIS PATHWAYS IN CANCER AND *ALZHEIMER'S* DISEASE

### 5.1 mTOR signaling pathway and Alzheimer's disease

mTOR (mammalian target of rapamycin), a serine/threonine protein kinase that centrally regulates cell metabolism, constitutively serves as the catalytic module inside two intracellular protein complexes, namely mTORC1 (mTOR Complex 1) and mTORC2 (mTOR Complex 2) (Wang, Jie, Joo, Ordureau, & Liu, 2017; Xu, Liu & Wei, 2014). With respect to structure, mTORC1 consists of three components, including mTOR, Raptor, and mLST8 (mTOR‐associated protein, LST8 homolog). The subunit Raptor is critical for substrate recognition and subcellular localization of mTORC1. Moreover, mLST8 facilitates the catalytic function of mTORC1 by stabilizing the kinase activity, playing a dispensable and auxiliary role (Crino, 2016). Similar to mTORC1, mTORC2 also includes both mTOR and mLST8 subunits. Nevertheless, Raptor is replaced by Rictor inside mTORC2, which analogously assists to recruit substrate and localize the kinase complex (Saxton & Sabatini, 2017).

As a key complex in regulating cellular response to nutrients, mTORC1 and its downstream pathways mediate multiple fundamental regulations of cells, including mRNA translation, metabolism, and protein homeostasis (Saxton & Sabatini, 2017). On the contrary, mTORC2 mainly serves to control cell survival and proliferation, via activating the Akt pathway (Zoncu, Efeyan & Sabatini, 2011). On the basis of its pro‐anabolic capability and central role in governing metabolic homeostasis, numerous evidence has demonstrated that inhibition of mTOR activity could lead to extended lifespan among mammalian species, implying a direct link between mTOR pathway and aging (Antikainen, Driscoll, Haspel, & Dobrowolski, 2017). Regarding the pathophysiological association between aging and *Alzheimer's* disease, the impact of mTOR in *Alzheimer's* disease has therefore been extensively investigated.

Transgenic overexpression of *mTOR* in murine brains culminates in cortical atrophy and neurodegeneration (Kassai, Sugaya, Noda, Nakao, & Maeda, 2014), while conditional knockout of *Tsc1* (TSC complex subunit 1), an mTOR inhibitor, has also successfully induced the neurodegenerative abnormalities in mouse brains, a phenocopy of AD pathophysiology (Carson, Nielen, Winzenburger, & Ess, 2012) (Table 2). At present, activation of mTOR has been recognized as a major event that causes AD pathogenesis (Yates, Zafar, Hubbard, Nagy, & Durant, 2013). Although the underlying mechanisms that contribute to hyperactivity of mTOR remain largely mysterious, cellular senescence, oxygen stress, and other unexpected insults that cause the activation of MAPK and PI3K/Akt pathways are regarded as probable mechanisms, which subsequently induces the activation of mTOR (Cai, Chen, He, Xiao, & Yan, 2015).

There are roughly two types of downstream mechanisms secondary to mTOR activation, known as autophagy‐dependent and autophagy‐independent pathways, respectively. Concerning autophagy‐dependent mechanism, activation of mTOR inhibits the kinase activity of ULK1 and thus blocks the initial formation of phagopores (Min et al., 2017). The downregulated autophagy subsequently results in reduced clearance of Aβ, elevated Aβ deposition, and eventually formation of Aβ plaques (Nixon, 2013). Moreover, the insufficient autophagy could also lower the clearance of misfolded tau proteins, leading to the accumulation of tau aggregates in the cytoplasm of neurons (Li et al., 2017). On the contrary, Aβ could also elevate mTOR activity through PRAS40 (proline‐rich Akt substrate 40 kDa)‐mediated mechanism and thus further dampens the autophagic response (Caccamo, Maldonado, Majumder, Medina, & Holbein, 2011). On the other hand, the autophagy‐independent pathway is also a vital mechanism that triggers both pathological formation of Aβ plaques and tau aggregates. The activation of mTOR elevates the expressions of β‐secretase and γ‐secretase with the involvement of AMPK and IGF‐1 (insulin‐like growth factor 1)‐mediated pathways. These enzymes serve to cleave APP and thus enhance Aβ production (Cai et al., 2015). With regard to tau, upregulation of mTOR facilitates the hyperphosphorylation on tau, via synergistically enhancing the activity of GSK‐3β (a kinase of tau protein) and suppressing PP2A (serine/threonine protein phosphatase 2A, a phosphatase of tau protein) (Wang, Yu, Miao, Wu, & Tan, 2014).

**Figure 4:**
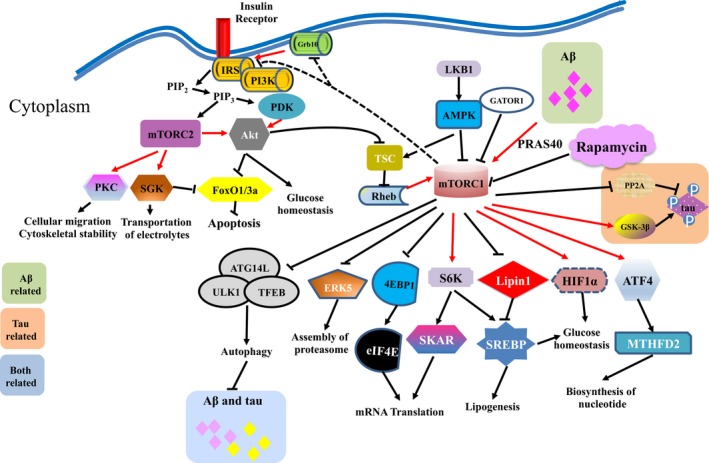
Core interactions between the mTOR pathway and *Alzheimer's* disease

Taken together, the aberrant activation of mTOR is involved in nearly every aspect of AD pathogenesis from the production toward the clearance of proteotoxins and is therefore regarded as a promising therapeutic target (Table 3) (Figure 4).

### 5.2 Endoplasmic reticulum (ER) stress signaling pathways

ER is a fundamental subcellular organelle responsible for the processing and transportation of intracellular proteins (Bhat, Chaudhary, Kumar, O'Malley, & Inigo, 2017). With respect to physiology, chaperones resident on the membrane of ER guarantees the accurate folding of newly assembled proteins. Those perfectly folded proteins will be subsequently transported to Golgi apparatus, while those unfolded proteins will be sent to proteolytic processes for recycling, including ubiquitin–proteasome system and autophagic machinery (Hetz & Saxena, 2017). The accumulated misfolded proteins will trigger ER stress as well as its corresponding cellular response called unfolded protein response (UPR), which is a protective action to sustain cell survival. Nevertheless, if the stress is too severe to be handled, apoptosis signaling will be subsequently activated and results in cell death instead (Hetz, 2012; Wang & Kaufman, 2014). Across mammalian species, three key ER stress sensors contribute to the initiation of UPR, including activating transcription factor 6 (ATF6), double‐stranded RNA‐activated protein kinase‐like ER kinase (PERK), and inositol‐requiring transmembrane kinase 1 (IRE1). Normal UPR enhances the expression of ER chaperones in order to accelerate the folding of raw proteins and helps transport the misfolded proteins to cytoplasm to alleviate the burden on ER, highlighting its central role in mediating protein homeostasis (Hetz & Mollereau, 2014; Xiang, Wang, Zhang, & Han, 2017).

It is well known that AD is a neurodegenerative disorder pathologically featuring the accumulation of misfolded proteins (Heneka, Carson, Khoury, Landreth, & Brosseron, 2015). As the ER is the main intracellular organelle responsible for the processing of proteins, the correlation between ER stress and AD has therefore drawn much attention. The heightened level of ER stress and downstream responses in AD‐affected regions has been confirmed by both in vivo and in vitro evidence (Remondelli & Renna, 2017). Conditional deletion of *Ire1*, a key component of the UPR, directly results in reduced cognitive impairment in rodent neurodegenerative models, which further confirms the promotive role by ER stress toward AD progression (Duran‐Aniotz, Cornejo, Espinoza, Ardiles, & Medinas, 2017) (Table 2). At present, while the initiation of ER stress in susceptible neurons remains unclear, genetic alterations, age‐related functional insufficiency, oxidative stress, or secondary to proteotoxic damage are assumed to be possible causes, which eventually lead to the impaired chaperone, calcium imbalance, and ER stress (Cai, Arikkath, Yang, Guo, & Periyasamy, 2016).

Mechanistic studies have implicated ER stress in impairing the function of ER protein BiP (ER luminal‐binding protein precursor), which binds to APP in the ER, guides its conformational modifications, and inhibits the production of Aβ. Increased stress in the ER diminishes the binding affinity of BiP to protein targets, disrupts the physiological folding of APP, and promotes its cleavage into Aβ. On the other hand, Aβ could also destroy the anchoring between ER and microtubules, which decreases the stability and aggravates the dysfunction of the ER system (Xiang et al., 2017). In regard to tau tangles, activated components of the UPR frequently co‐localize with hyperphosphorylated tau. Further mechanistic analyses have shown that phosphorylation of PERK activates its downstream kinase GSK‐3β, which in turn leads to the hyperphosphorylation of tau and facilitates the formation of tau tangles (Roussel, Kruppa, Miranda, Crowther, & Lomas, 2013). Meanwhile, due to the excessive burden on the ER, the UPR could also trigger apoptotic death of affected neurons, which is a vital mechanism of neuronal death in AD‐affected regions (Li, Yu, Jiang, & Tan, 2015; Xiang et al., 2017) (3; 5).

**Figure 5:**
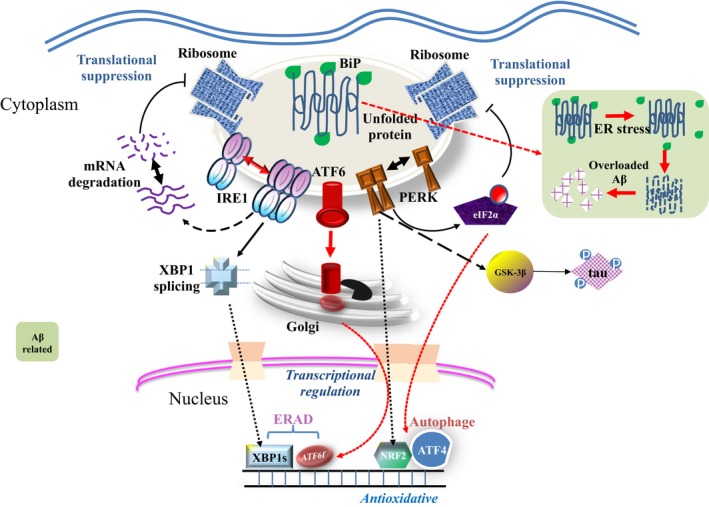
Pathological linkage between UPR and *Alzheimer's* disease

### 5.3 Other proteostatic systems in cancer and Alzheimer's disease

Apart from the aforementioned pathways, there are still other proteostatic systems that participate in the development of cancer and *Alzheimer's* disease, such as molecular chaperones and mitochondrial homeostasis (Kim, Hipp, Bracher, Hayer‐Hartl, & Hartl, 2013; Musci, Hamilton & Miller, 2017). Molecular chaperones are proteins that function to assist refolding of misfolded proteins or degradation of damaged proteins, which are mainly constituted by heat‐shock proteins (HSPs) (Kim et al., 2013). Different molecular chaperones exert either positive or negative impacts on the pathogenesis of AD. For instance, HSP40 and HSP70 are able to inhibit the formation of Aβ plaques by refolding the excessive Aβ peptides into soluble forms, while HSP90 facilitates the pathogenic folding of tau protein into neurotoxic tangles, thus promoting AD progression (Ciechanover & Kwon, 2017). In terms of neoplastic transformation, the majority of chaperones are upregulated in cancerous tissues displaying oncogenic roles, especially members from the HSP family, such as HSP27, HSP70, and HSP90. Taking HSP70 as an example, it plays essential roles in triggering the growth, metastasis, and chemo‐resistance of breast cancer, which could be greatly inhibited by the knockout of *Hsp70* (Calderwood & Murshid, 2017). This evidence implies the potential of chaperone‐targeted therapy in both AD and cancer therapeutic strategies. To this end, HSP90‐specific chaperone inhibitors, such as 17‐AAG, have been used to attenuate Aβ‐induced synaptic toxicity (Ansar, Burlison, Hadden, Yu, & Desino, 2007; Chen, Wang, Liu, Li, & Xue, 2014; Solit & Chiosis, 2008). A4, as a nontoxic C‐terminal inhibitor of HSP90, also has been established to induce Hsp90 overexpression and permeate the blood–brain barrier, and its functions have been indicated in protecting neurons against Aβ‐induced toxicity (Ansar et al., 2007).

Recent studies have further revealed that both natural aging and AD are associated with a significant decline in the omega‐3 polyunsaturated fatty acid docosahexaenoic acid (DHA), and thus, treatment of DHA has been proposed as a potential strategy for AD therapy (Grimm, Zimmer, Lehmann, Grimm, & Hartmann, 2013). 2‐hydroxy‐DHA (HDHA), a docosahexaenoic acid (DHA) derivate, could restore cognitive function in an AD transgenic mouse model (Arsenault, Julien, Tremblay, & Calon, 2011). HDHA also prevents both cytoskeleton perturbations and apoptosis induced by Aβ‐soluble oligomers. In a mechanical manner, HDHA exposure not only induced UPR activation, indicated by the upregulation of Bip protein and phosphorylation status of eIF2a, but also upregulated proteins involved in initiating autophagy, such as beclin‐1 and Atg proteins (Atg3, Atg5, Atg12, and Atg7) (Torres, Marcilla‐Etxenike, Fiol‐deRoque, Escribá, & Busquets, 2015). Thus, these findings suggest a possible strategy to alleviate AD by manipulating UPR and autophagy pathways through HDHA administration.

### 5.4 Clinical and pharmaceutical implications

So far, mTOR inhibitors have only been approved against malignant tumors, including rapamycin analog temsirolimus for kidney cancer and everolimus for neuroendocrine tumors (Gajate, Martínez‐Sáez, Alonso‐Gordoa, & Grande, 2017; Ghidini, Petrelli, Ghidini, Tomasello, & Hahne, 2017). Nevertheless, due to limited efficacy and high resistant rate, these drugs may be substituted by second‐generation mTOR inhibitors that feature higher effectiveness and less refractoriness (Saxton & Sabatini, 2017). With regard to *Alzheimer's* disease, although rapamycin and temsirolimus demonstrate great anti‐AD effects according to both preclinical and clinical evidence (Jiang et al., 2014; Maiese, 2014), currently there are no official approvals for their clinical application yet.

Considering the tumorigenic role of ER stress in cancer development, many targeted chemicals exhibit antitumor effects via inhibiting key components of the UPR (Cubillos‐Ruiz, Bettigole & Glimcher, 2017). Specifically, IRE1α kinase inhibitor KIRA6 which inhibits the activity of IRE1α to halt the initiation of UPR and its protective manner to cancer cells. Moreover, GSK2606414 and GSK2656157 also suppress the malignant traits of various tumors by inactivating another UPR initiator PERK (Cubillos‐Ruiz et al., 2017; Ghosh, Wang, Wang, Perera, & Igbaria, 2014). Regarding *Alzheimer's* disease, PERK inhibitor GSK2606414 likewise attenuates the severity of AD through decreasing the accumulation of misfolded plaques and tangles (Radford, Moreno, Verity, Halliday, & Mallucci, 2015). However, all of these evidences are based on preclinical data, and thus, clinical trials are urgently needed (Table 4).

### 5.5 Dysregulation of protein homeostasis pathways in different neural cells

The “cholinergic hypothesis” was initially presented 20 years ago, and further studies suggest that a dysfunction of cholinergic neurons in the brain contributes to the cognitive decline observed in AD due to their role in memory (Cuello & Bruno, 2007; Schliebs & Arendt, 2011). This hypothesis established cholinergic neurons as more vulnerable to β amyloid plaque formation, and however, the underlying reason has not been well defined (Cuello & Bruno, 2007). Recent studies have suggested that aging‐related cognitive deficits may be due to impairments of cholinergic function rather than specific cell loss (Cuello & Bruno, 2007; Schliebs & Arendt, 2011). Studies also demonstrate that nerve growth factor (NGF) is specifically implicated in protection and maintenance of cholinergic neurons in the basal forebrain (Bruno & Cuello, 2006). Due to the fact that basal forebrain cholinergic neurons are highly dependent on the constant internal supply of NGF, if the supply of NGF is interrupted, cholinergic atrophy could begin to occur in these neurons and change their phenotype. However, this supply could be interrupted if there is a failure in the protease cascade and the precursor proNGF cannot be converted to NGF. Thus, the pathways responsible for both the maturation and degradation of NGF could cause cholinergic neurons to become more vulnerable (Cuello & Bruno, 2007). This failure of NGF stimulation leads to the progressive atrophy of basal forebrain cholinergic neurons, which in turn contributes to AD‐related learning and memory deficiency. Emerging evidence also demonstrated that β amyloid proteins bind to cholinergic neurons and physically inhibit choline acetyltransferase (ChAT) activity in culture conditions when treated with oligomers of β amyloid (Nunes‐Tavares, Santos, Stutz, Brito‐Moreira, & Klein, 2012). On the other hand, the neurofibrillary tangles seem to increase within the basal forebrain cholinergic complex in patients with AD as well. Taken together, these findings indicate that the dysregulation of protein homeostasis contributes to making cholinergic neurons more susceptible to Aβ or tau aggregation, and therefore, Aβ or tau aggregation could further disturb cholinergic neuron functions to promote AD.

A previous study has also demonstrated that the reactive glial shows increased immunoproteasome activity in AD to clear Aβ accumulation (Orre, Kamphuis, Dooves, Kooijman, & Chan, 2013), but the underlying mechanism is not well defined. More interesting, recent studies found a novel microglia cell type associated with neurodegenerative diseases (DAM cells) using single‐cell RNA‐seq technology, which displayed a unique capability to clear Aβ (Keren‐Shaul, Spinrad, Weiner, Matcovitch‐Natan, & Dvir‐Szternfeld, 2017). Furthermore, another group also found glial cells in patients with AD bearing TREM2 variants, and *Trem2*‐deficient mice have a greater abundance of autophagic vesicle accumulation and an AD‐like pathology (Ulland, Song, Huang, Ulrich, & Sergushichev, 2017). In a mechanical manner, they reported that these specific glial cells are activated sequentially by both Trem2‐independent and Trem2‐dependent pathways, which involved in mTOR‐mediated autophagy and metabolic biosynthetic pathways (Ulland et al., 2017). These findings together suggest that the disturbance of proteostasis in glial cells mediated by the TREM2‐mTOR axis may lead to further Aβ accumulation.

## 6 SUMMARY

In general, mTOR cascade and endoplasmic reticulum serve as the upstream elements inside the proteostatic system, which chiefly determine the synthesis and conformation of target proteins while ubiquitin–proteasome system and autophagy act as main scavengers of misfolded or excessive proteins, regarded as the final step of proteostatic machinery. The mutual interactions and interplay between upstream and downstream components form a complex regulatory circuit for the homeostasis of proteins.

Analogous to other neurodegenerative disorders, the main cause of *Alzheimer's* disease is the accumulation of misfolded proteins, known as Aβ plaques and tau aggregates. Therefore, dysregulation of proteostasis contributes to the accumulation of proteotoxins in *Alzheimer's* disease. The aberrant circuit triggers the progression of AD by either enhancing the production or decreasing the clearance of pathogenic tau and Aβ, eventually leading to the pathological transformation in neurons. In view of the critical and comprehensive role that protein homeostasis‐governing pathways have played in the pathogenesis of AD, targeted therapy may have great potential to solve the current therapeutic dilemma against AD.

## CONFLICT OF INTEREST

None declared.
